# Supplementary figures and images for: Self-assembly of Hyaluronic Acid-Cu-Quercetin flavonoid nanoparticles: synergistic chemotherapy to target tumors
Source: PeerJ. 2023 Aug 28;11:e15942. doi: 10.7717/peerj.15942 (PMC10470444; doi:10.7717/peerj.15942)

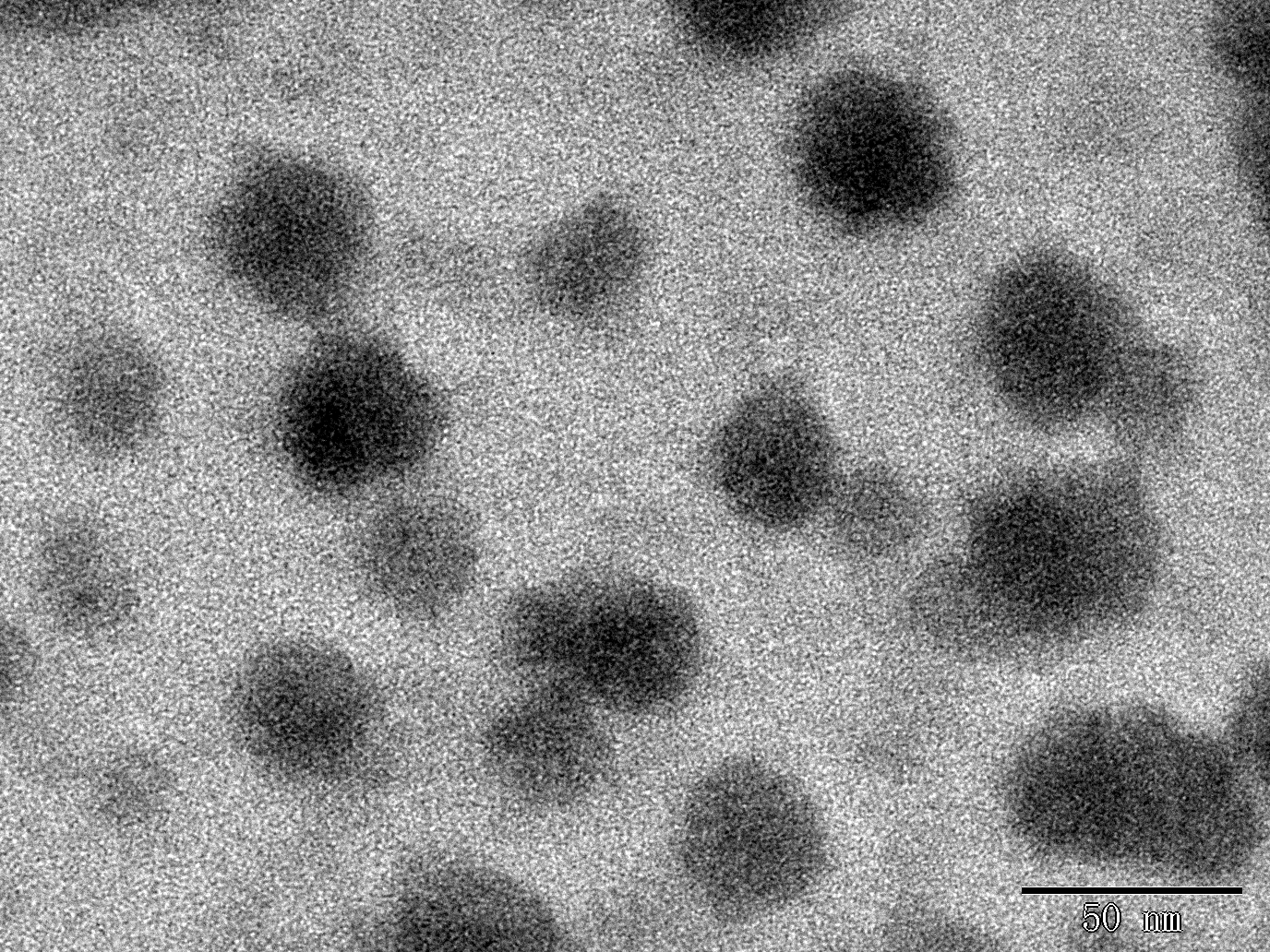

Supplement: Supplemental Information 1 [file peerj-11-15942-s001.zip › Fig 1a/50 nm.tif]

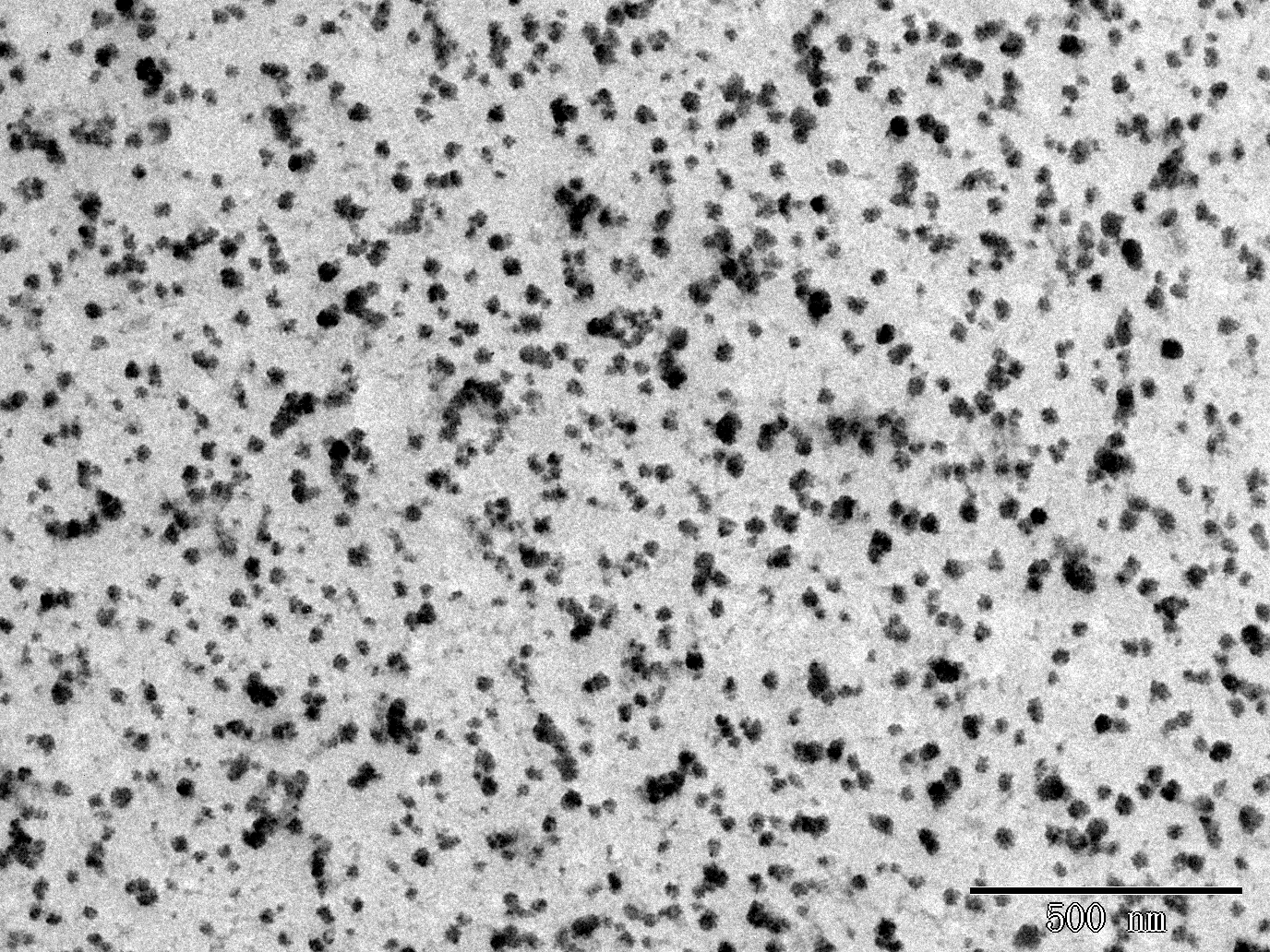

Supplement: Supplemental Information 1 [file peerj-11-15942-s001.zip › Fig 1a/500 nm.tif]

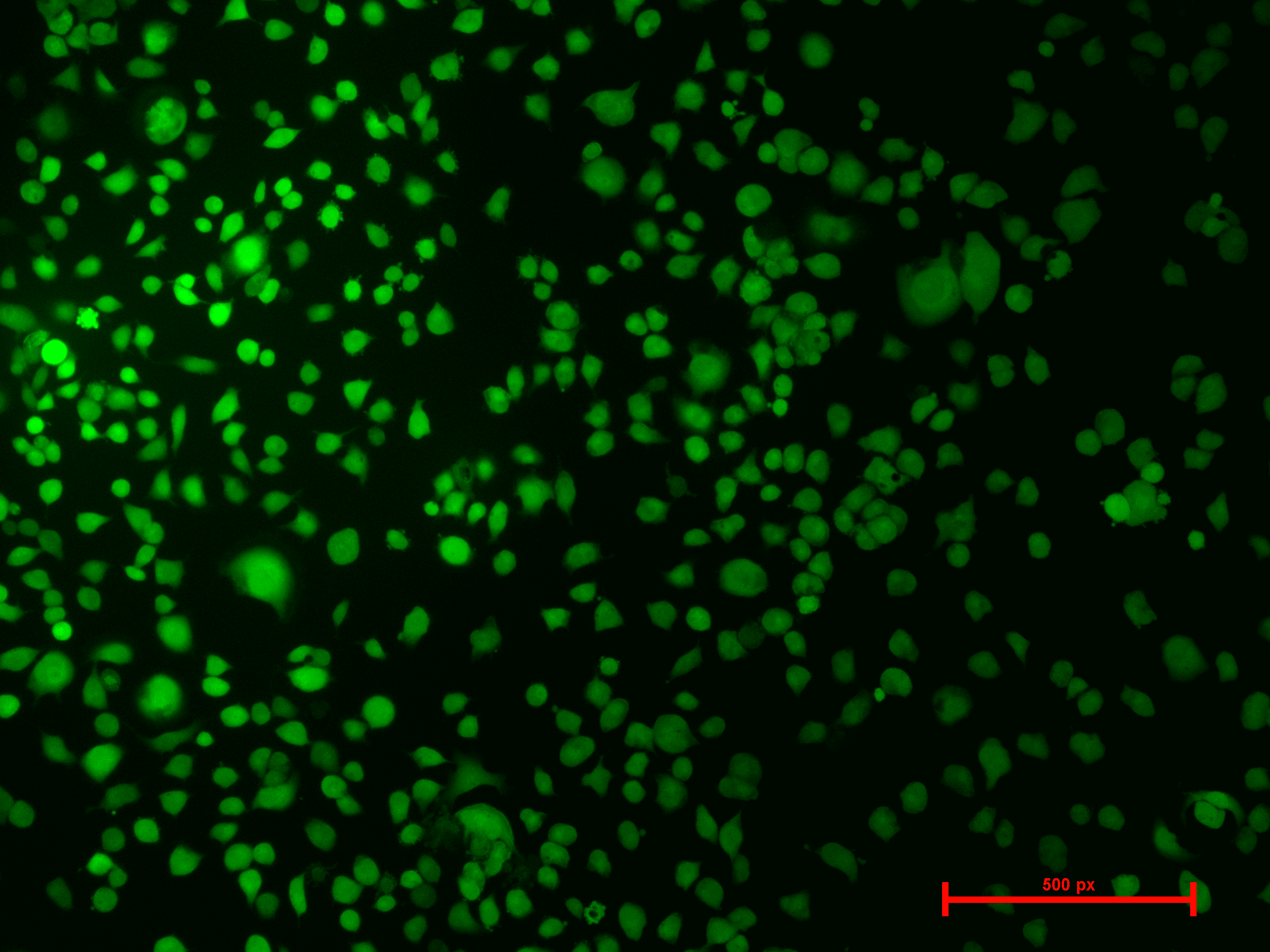

Supplement: Supplemental Information 1 [file peerj-11-15942-s001.zip › Fig 3b/HCQ+H2O2.tif]

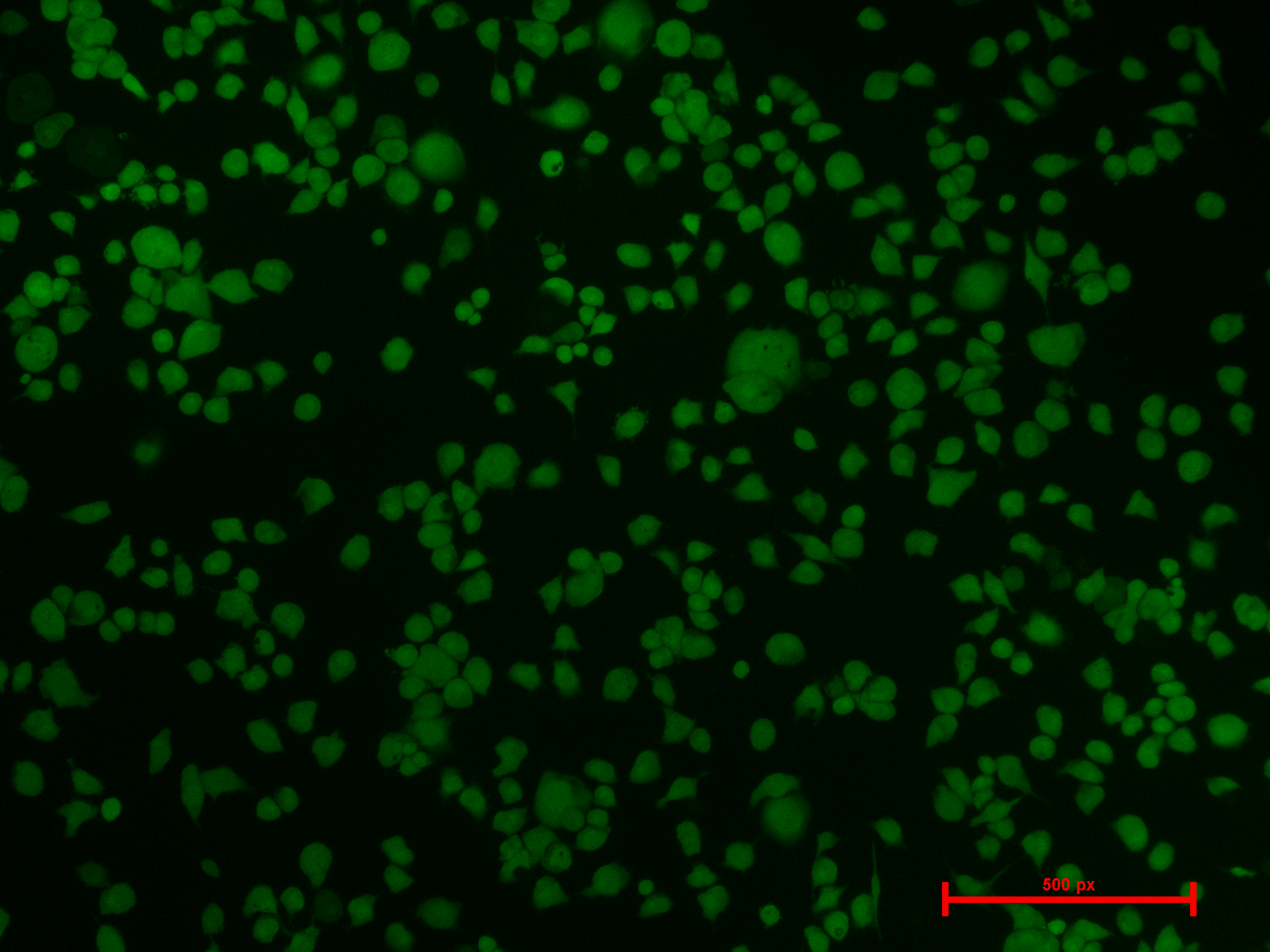

Supplement: Supplemental Information 1 [file peerj-11-15942-s001.zip › Fig 3b/HCQ.tif]

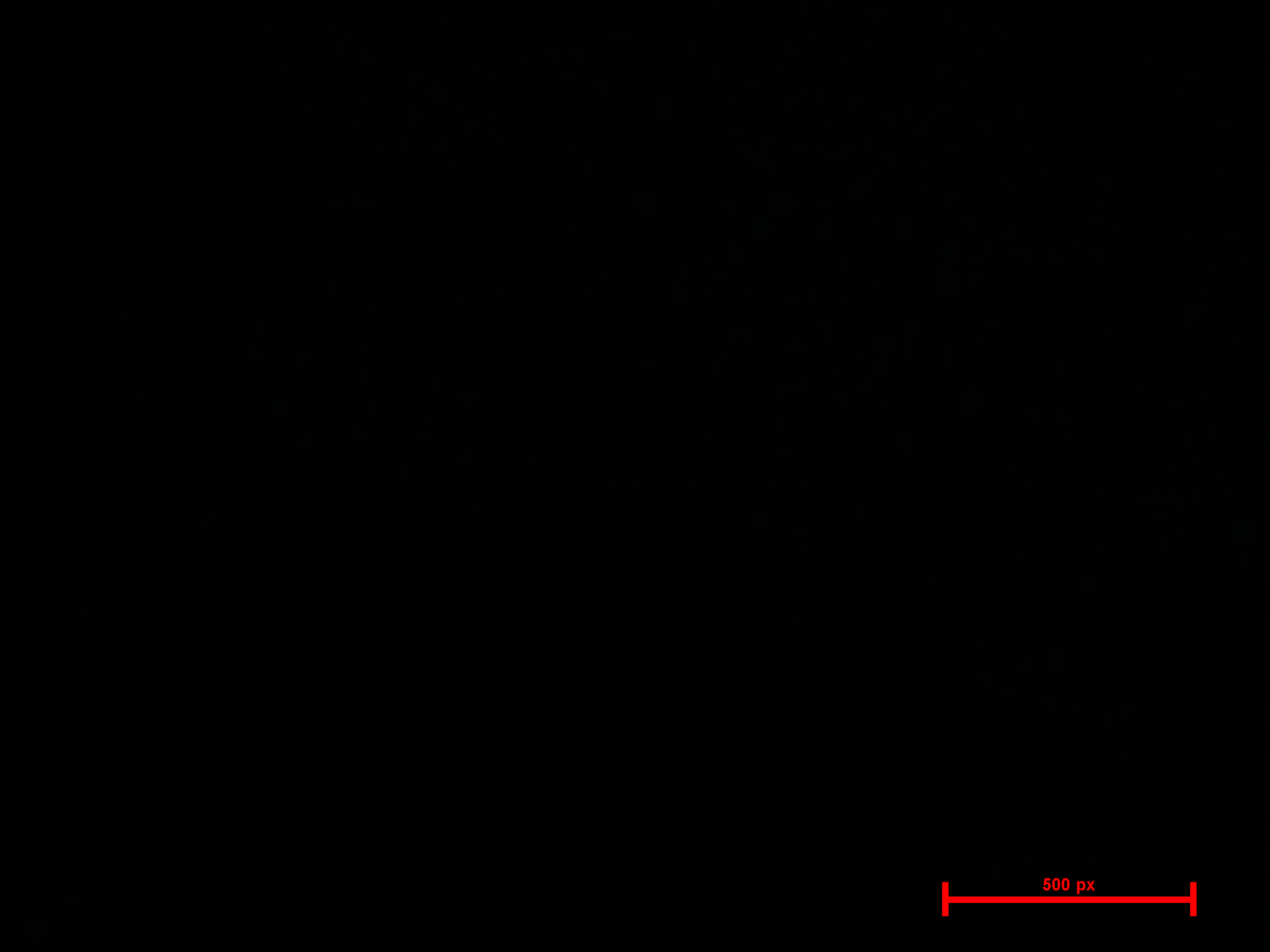

Supplement: Supplemental Information 1 [file peerj-11-15942-s001.zip › Fig 3b/PBS.tif]

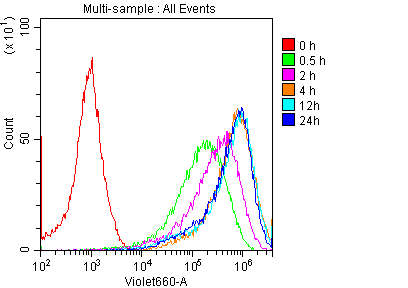


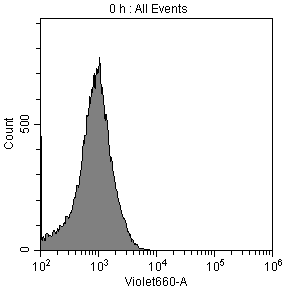


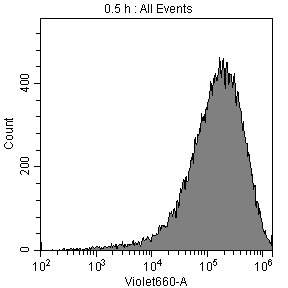


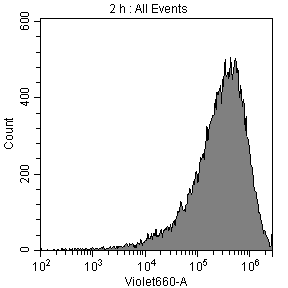


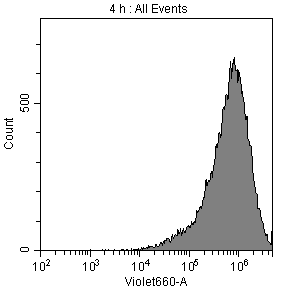


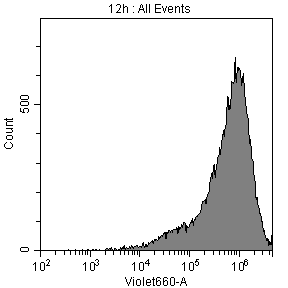


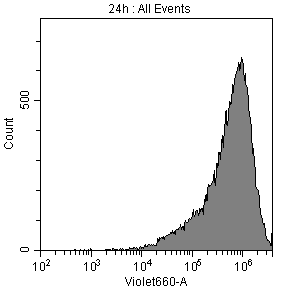


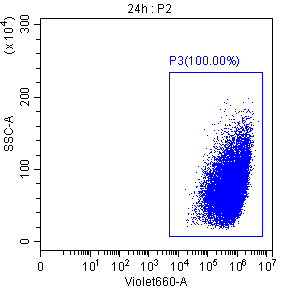

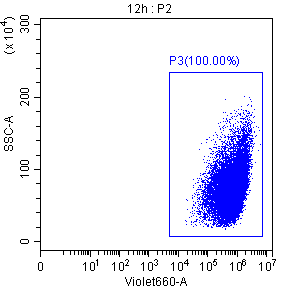

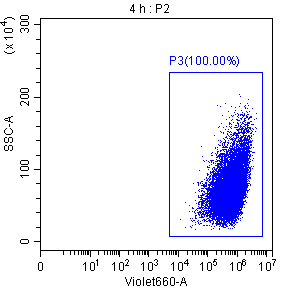

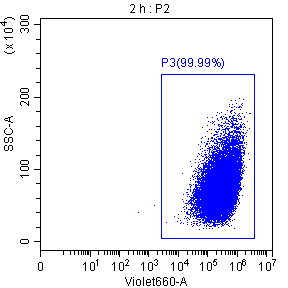

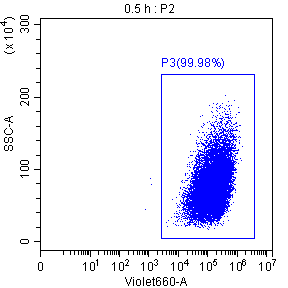

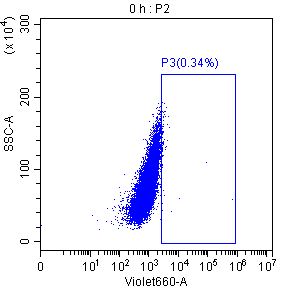

Supplement: Supplemental Information 1 [file peerj-11-15942-s001.zip › Fig 4a/cellular uptake.docx]

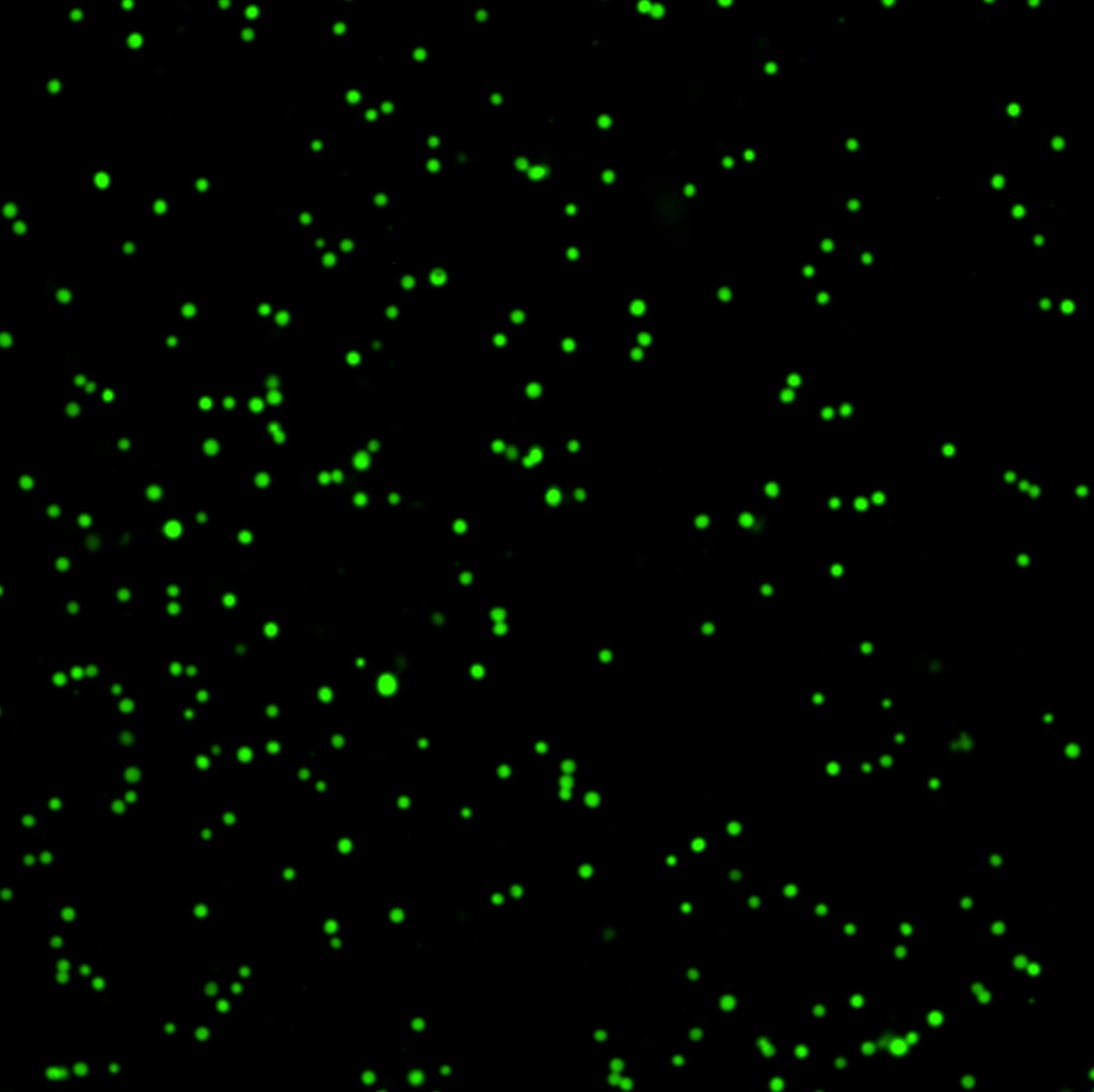

Supplement: Supplemental Information 1 [file peerj-11-15942-s001.zip › Fig 4d/HCQ+H2O2-AM.tif]

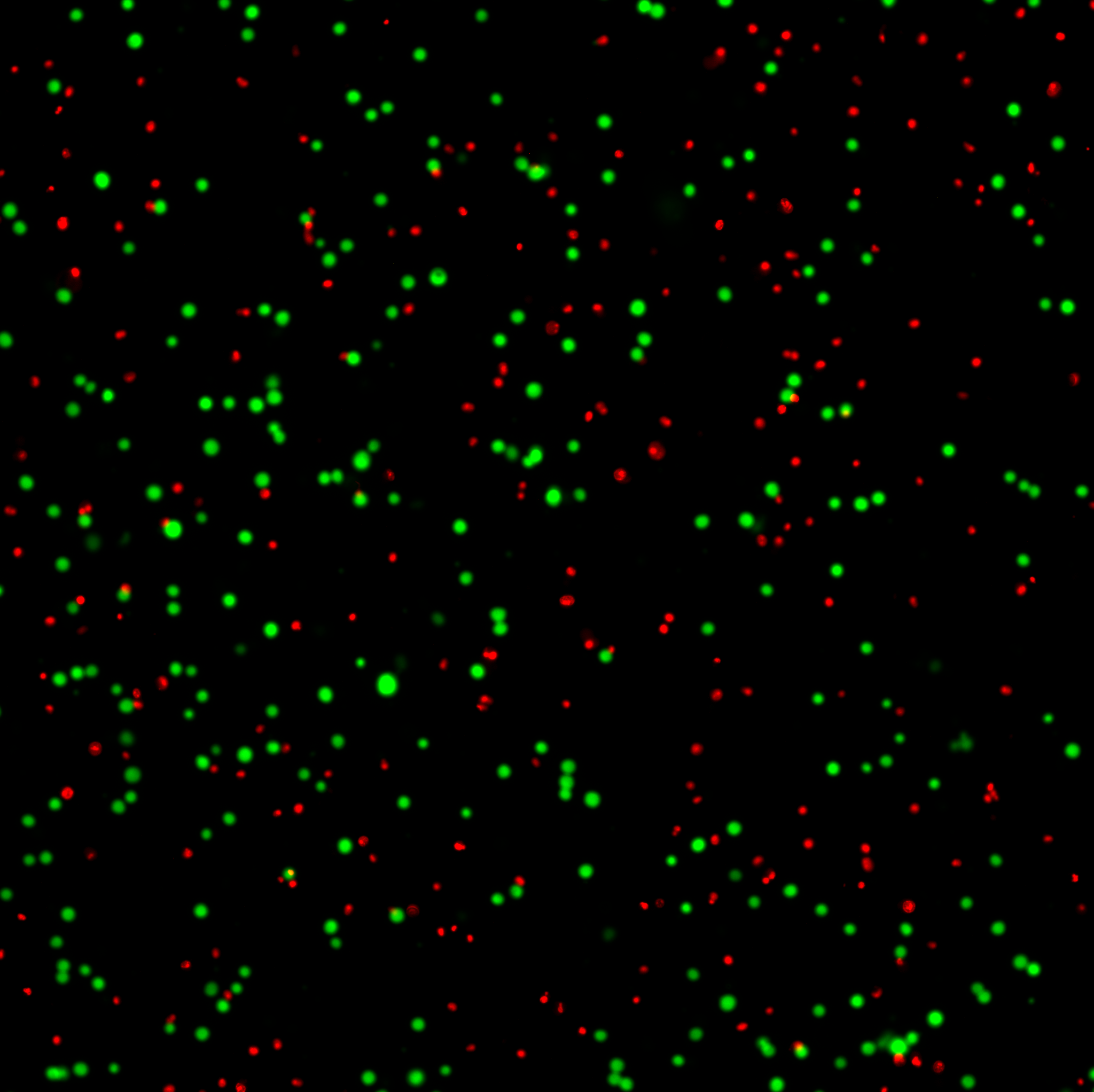

Supplement: Supplemental Information 1 [file peerj-11-15942-s001.zip › Fig 4d/HCQ+H2O2-Overlay.tif]

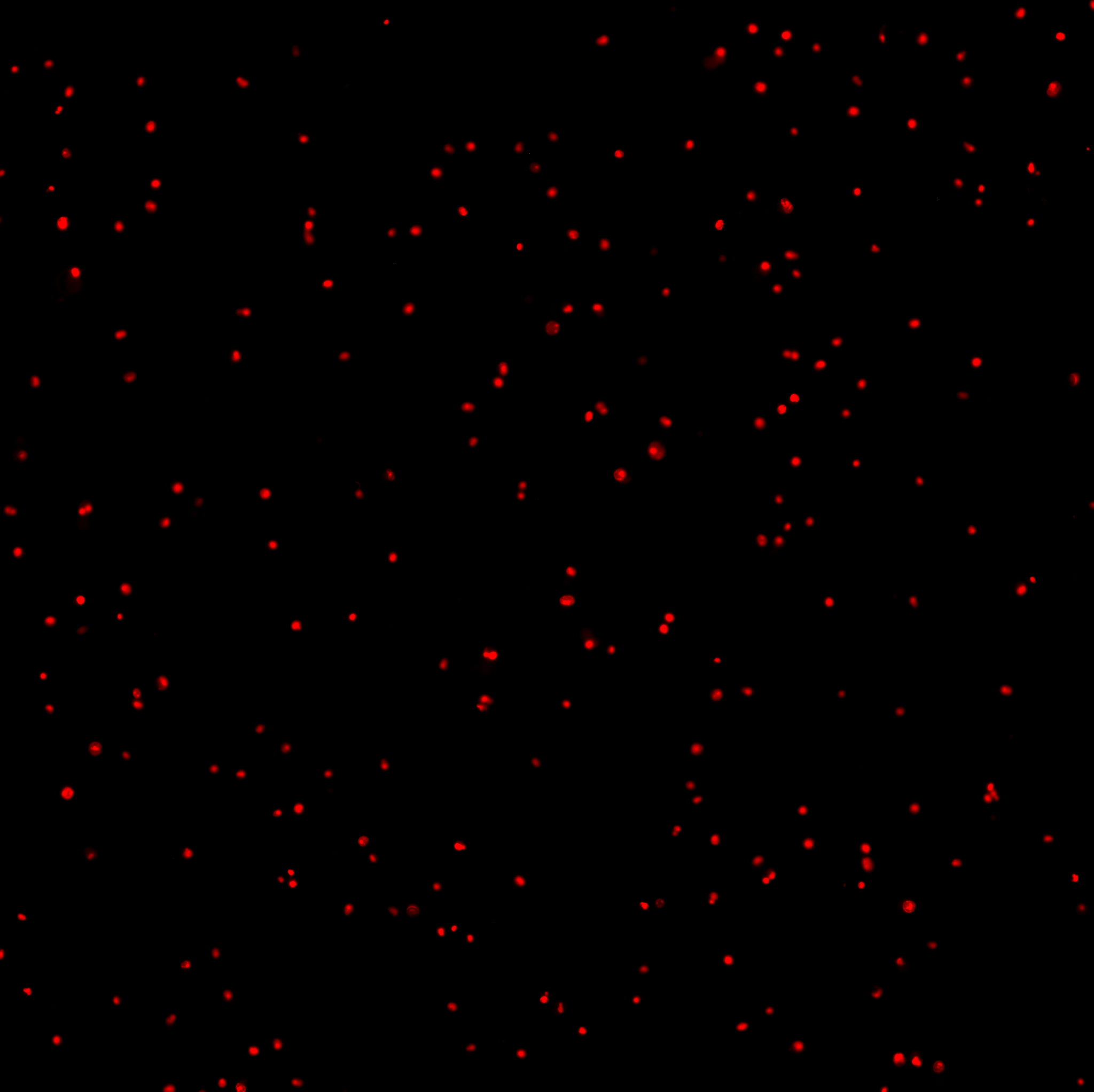

Supplement: Supplemental Information 1 [file peerj-11-15942-s001.zip › Fig 4d/HCQ+H2O2-PI.tif]

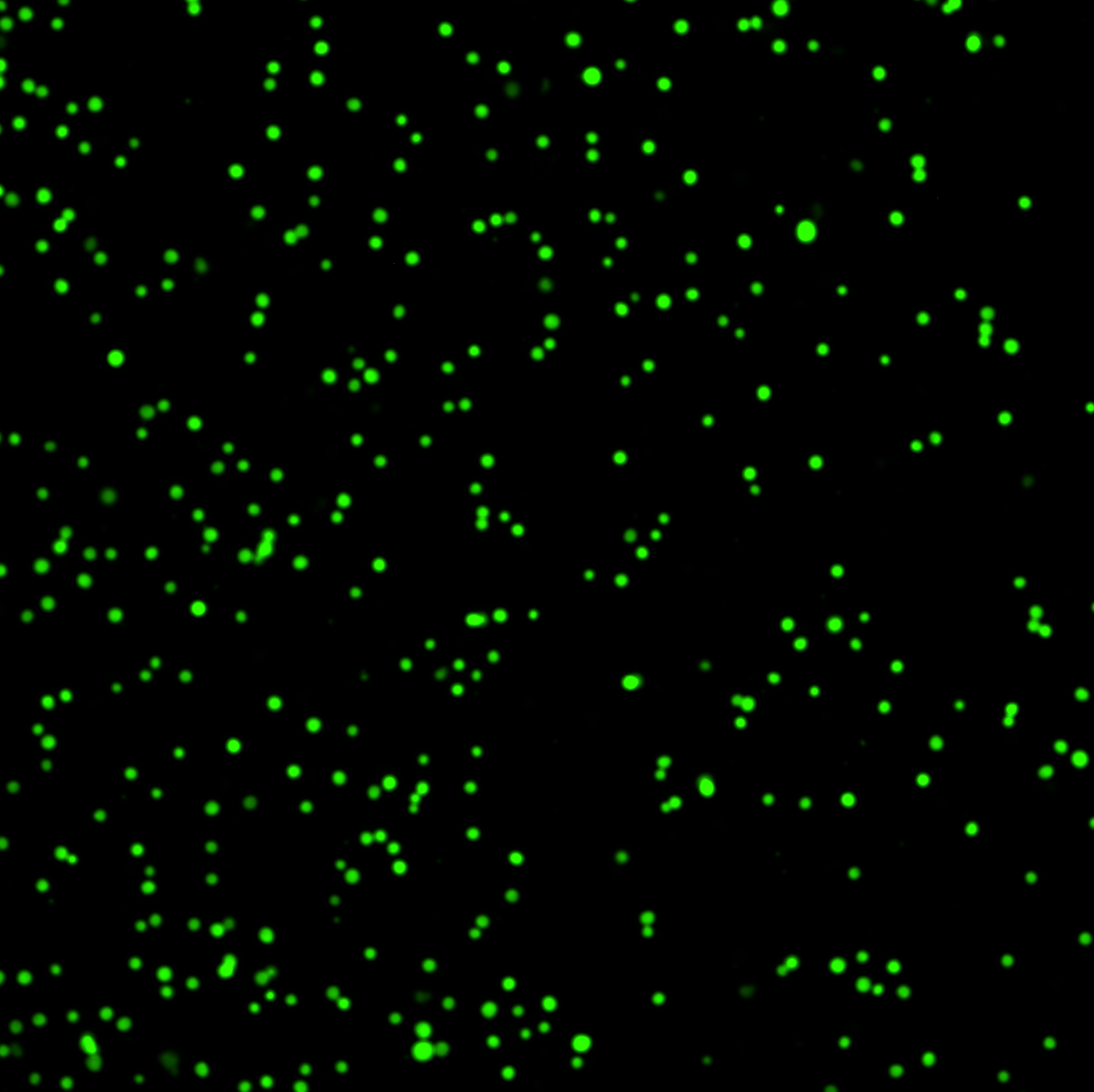

Supplement: Supplemental Information 1 [file peerj-11-15942-s001.zip › Fig 4d/HCQ-AM.tif]

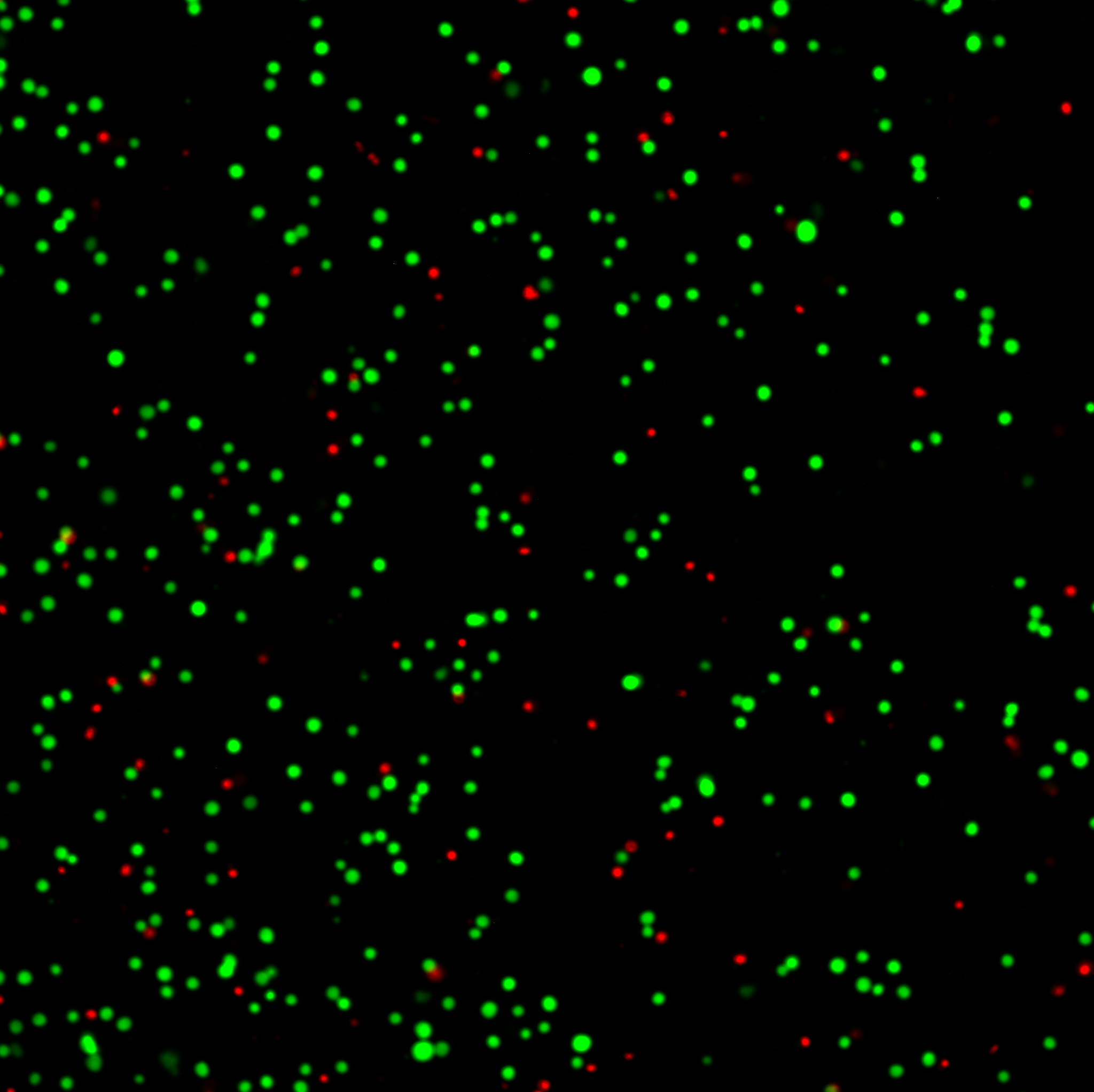

Supplement: Supplemental Information 1 [file peerj-11-15942-s001.zip › Fig 4d/HCQ-Overlay.tif]

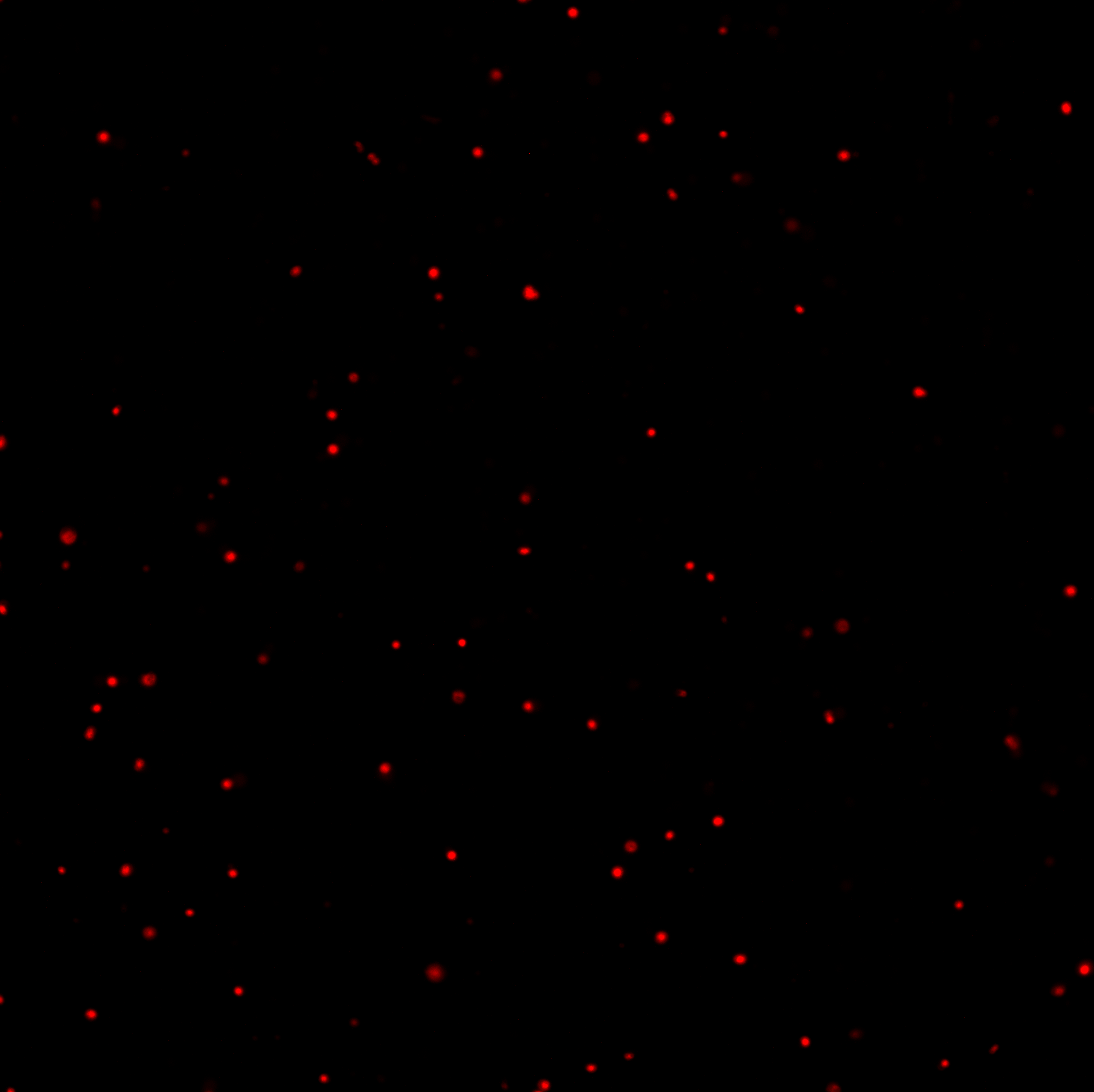

Supplement: Supplemental Information 1 [file peerj-11-15942-s001.zip › Fig 4d/HCQ-PI.tif]

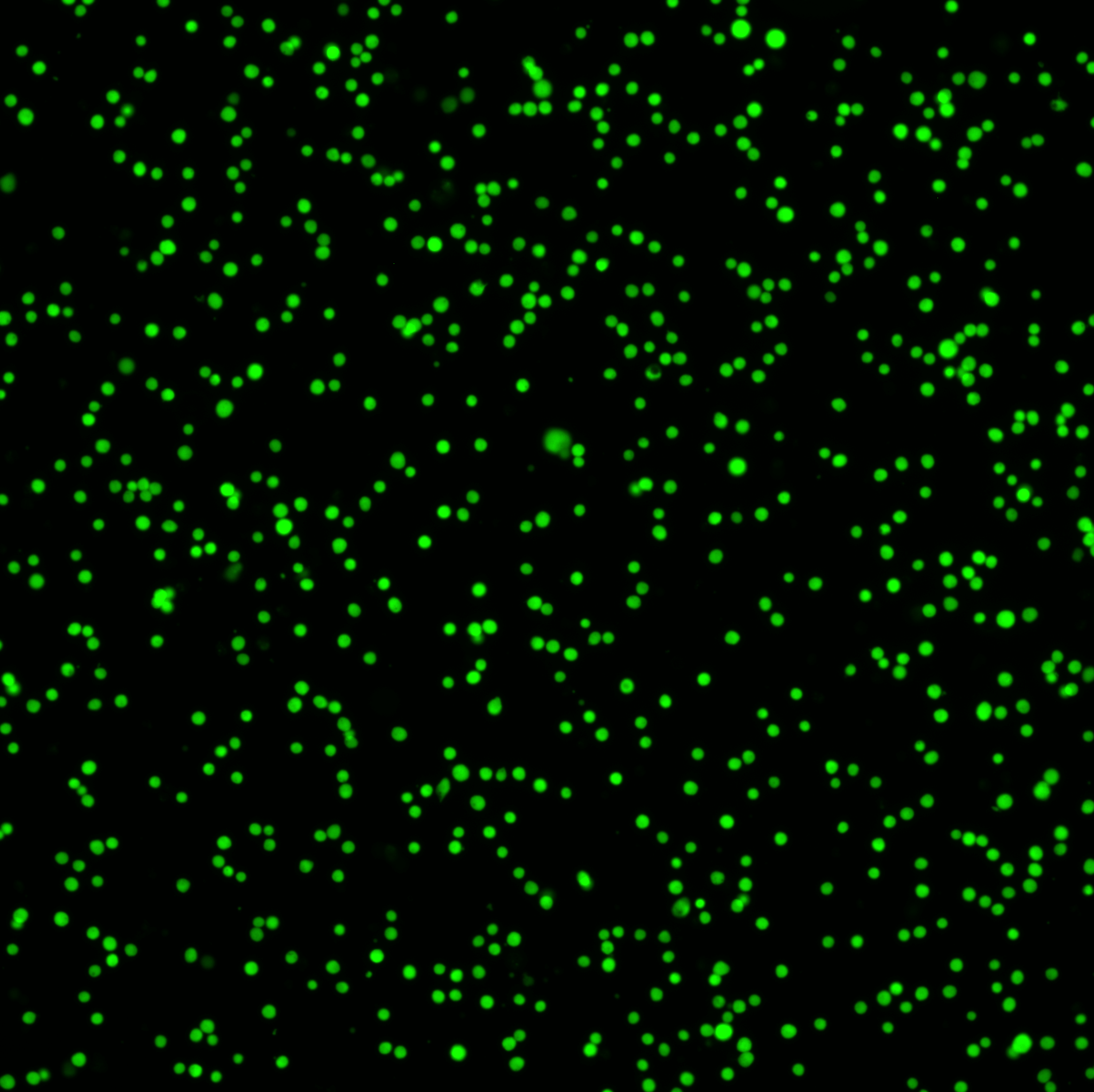

Supplement: Supplemental Information 1 [file peerj-11-15942-s001.zip › Fig 4d/PBS-AM.tif]

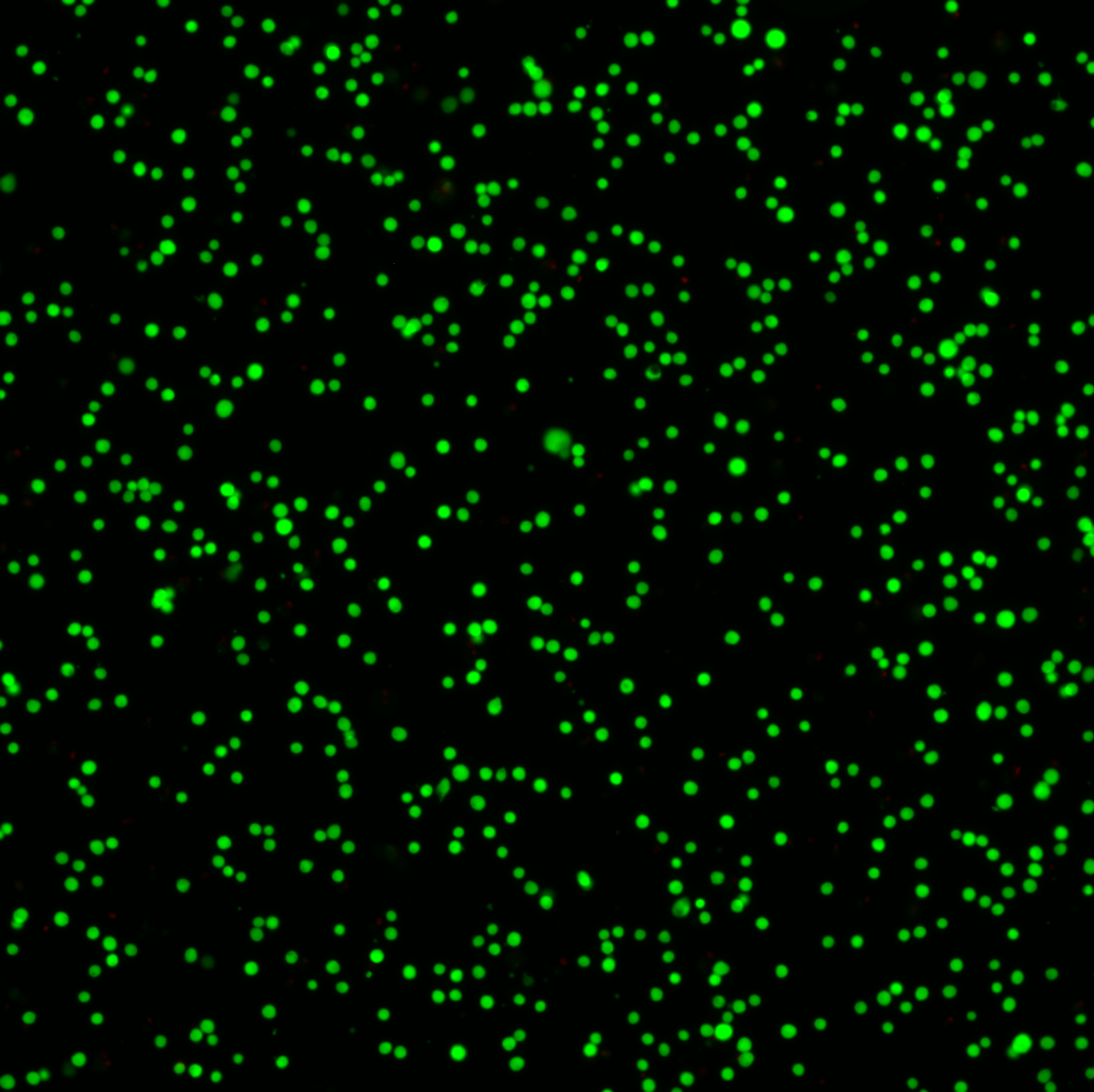

Supplement: Supplemental Information 1 [file peerj-11-15942-s001.zip › Fig 4d/PBS-Overlay.tif]

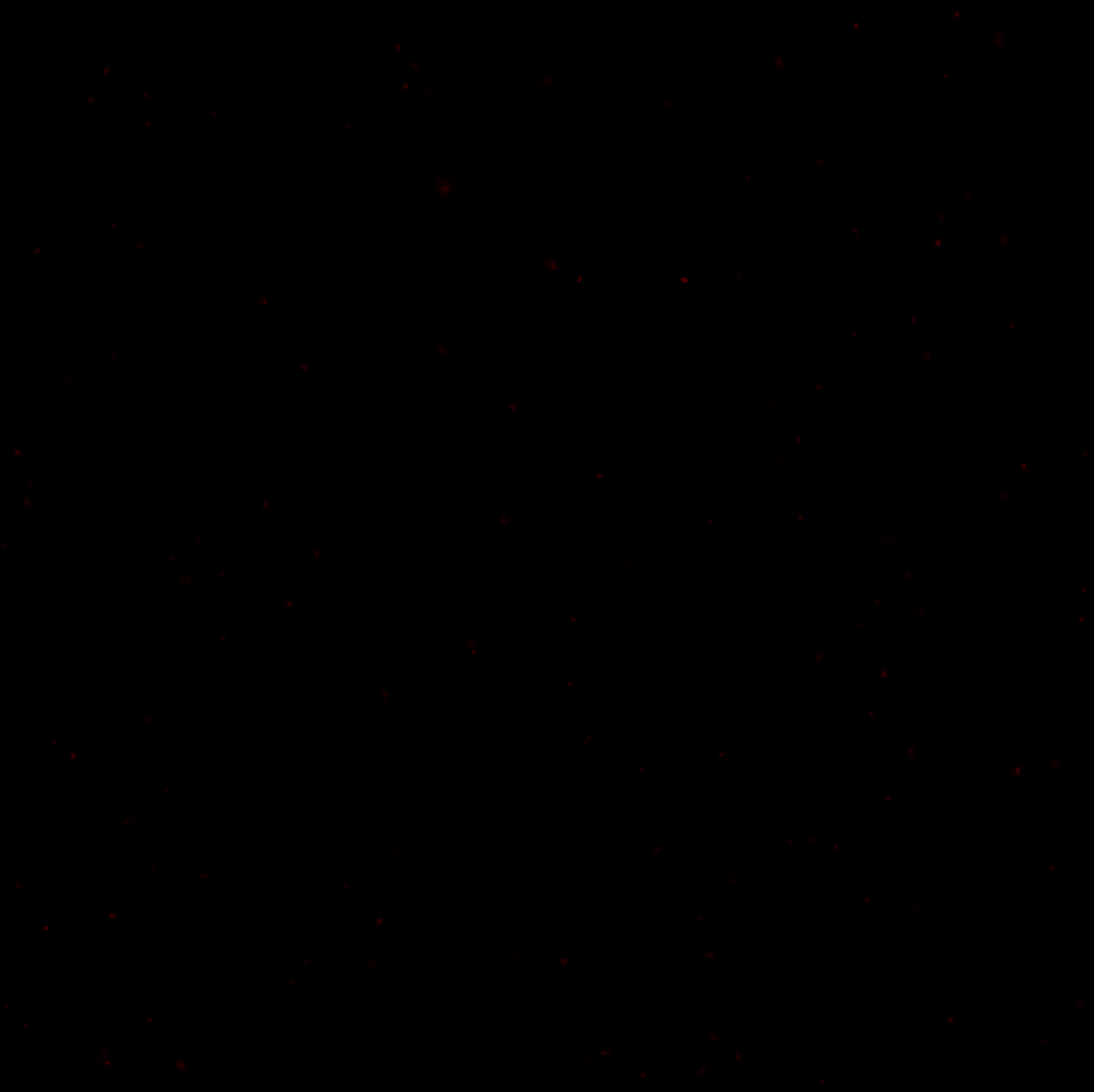

Supplement: Supplemental Information 1 [file peerj-11-15942-s001.zip › Fig 4d/PBS-PI.tif]
